# Gender Differences in Adolescent Sleep Disturbance and Treatment Response to Smartphone App–Delivered Cognitive Behavioral Therapy for Insomnia: Exploratory Study

**DOI:** 10.2196/22498

**Published:** 2021-03-23

**Authors:** Sophie H Li, Bronwyn M Graham, Aliza Werner-Seidler

**Affiliations:** 1 Black Dog Institute University of New South Wales Randwick Australia; 2 School of Psychology University of New South Wales Kensington Australia

**Keywords:** insomnia, gender differences, adolescents, sleep disturbance, sleep quality, sleep, gender, digital interventions

## Abstract

**Background:**

Insomnia and sleep disturbance are pervasive and debilitating conditions affecting up to 40% of adolescents. Women and girls are at greater risk of insomnia, yet differences in treatment responsiveness between genders have not been adequately investigated. Additionally, while women report greater symptom severity and burden of illness than men, this discrepancy requires further examination in adolescents.

**Objective:**

The purpose of this study was to examine gender differences in sleep symptom profiles and treatment response in adolescents.

**Methods:**

Digital cognitive behavioral therapy for insomnia (CBT-I) treatment responsiveness, as indexed by changes in Insomnia Severity Index (ISI) and Global Pittsburgh Sleep Quality Index (PSQI) scores, was compared in boys and girls (aged 12-16 years; N=49) who participated in a pilot evaluation of the Sleep Ninja smartphone app. Gender differences in self-reported baseline insomnia symptom severity (ISI), sleep quality (PSQI), and sleep characteristics derived from sleep diaries were also examined.

**Results:**

Compared with boys, we found that girls reported greater symptom severity (*P*=.04) and nighttime wakefulness (*P*=.01 and *P*=.04) and reduced sleep duration (*P*=.02) and efficiency (*P*=.03), but not poorer sleep quality (*P*=.07), more nighttime awakenings (*P*=.16), or longer time to get to sleep (*P*=.21). However, gender differences in symptom severity and sleep duration were accounted for by boys being marginally younger in age. Treatment response to CBT-I was equivalent between boys and girls when comparing reductions in symptom severity (*P*=.32); there was a trend showing gender differences in improvements in sleep quality, but this was not statistically significant (*P*=.07).

**Conclusions:**

These results demonstrate the presence of gender differences in insomnia symptoms and severity in adolescents and suggest further research is required to understand gender differences in insomnia symptom profiles to inform the development of gender-specific digital interventions delivered to adolescents.

## Introduction

Insomnia, defined as difficulty initiating or maintaining sleep with an associated impairment in daytime functioning [1], is a pervasive problem affecting 4%-18.5% of adolescents, in addition to 40% experiencing subthreshold symptoms and insufficient sleep [2-5]. Insomnia and disturbed and insufficient sleep impact adolescents’ academic, social, emotional, and behavioral development [6]. Difficulties with sleep also often accompany mental health disorders, such as depression, and contribute to their maintenance and burden of illness [7]. Moreover, there is a well-established link between sleep disturbance and the onset of depression in adolescents, emphasizing the need for effective interventions to ensure adequate sleep duration and quality in this population [8-10].

The exact cause of insomnia has not yet been identified but is likely to involve complex interactions between genetic, neurological, behavioral, cognitive, and emotional factors (eg, [11,12], particularly during adolescence [13]. One factor identified to be a likely contributing candidate is gender. Girls and women are 50% more likely to develop insomnia [14,15] compared with adolescent boys and men. One recent large-scale study examining insomnia symptoms in boys and girls aged 6-17 years found that pubertal maturation in girls was associated with increased prevalence and severity of insomnia symptoms, indicating that gender discrepancies in insomnia emerge at puberty [16]. Women also report greater symptom severity and perceived burden of illness [17] and more difficulty falling asleep and nighttime wakefulness than men [18]. Only one study has investigated gender discrepancies in sleep characteristics in adolescents. This study recruited a large cohort of more than 10,000 Norwegian adolescents with a mean age of 17 years and found that girls reported taking longer to get to sleep (sleep onset latency [SOL]: 51 minutes vs 43 minutes), experienced greater durations of wakefulness after sleep onset (wake after sleep onset [WASO]: 17 minutes vs 12 minutes), and reported a greater perceived need for sleep (8 hours 43 minutes vs 8 hours 26 minutes) [4].

Cognitive behavioral therapy for insomnia (CBT-I) is the gold-standard psychological treatment for insomnia and is effective for adults [19-22] and adolescents [23]. CBT-I consists of two main components. The first is cognitive therapy, which provides the individual with strategies to reevaluate catastrophic beliefs about sleep and manage sleep-inhibiting cognitive processes, such as worry and rumination. The second is the behavioral component, which encourages the application of behaviors associated with improved sleep, such as a consistent sleep routine and the elimination of daytime naps (for review, see [1]). CBT-I can be delivered in its traditional face-to-face format [20,24] or digitally as a web-based program [23] or smartphone app [25]. Face-to-face and digitally delivered CBT-I have been found to be equally beneficial [23]. However, digitally delivered CBT-I has the benefit of addressing several barriers to treatment identified in adolescents, such as accessibility and affordability, and allowing a degree of privacy to reduce concerns related to stigma, making digital delivery a particularly good treatment option for this age group. Regardless of delivery format, there is significant variability between individuals in treatment response. One potential source of treatment response variability may be the aforementioned gender differences in sleep symptom profiles. However, despite well-documented gender differences in insomnia prevalence, gender differences in insomnia treatment *responsiveness* have not been adequately investigated in either adults or adolescents. Specifically, only 2 studies have intentionally investigated the contribution of gender to treatment outcomes. One study examined the extent to which several demographic factors, including income, race, gender, age, and education, moderated treatment response in 358 adults with insomnia [26]. Neither gender nor any of the remaining demographic factors were significant moderators of CBT-I treatment response as measured by the Insomnia Severity Index (ISI). In a smaller study, Lami et al [27] investigated differential CBT-I treatment response in adult men (n=13) and women (n=15) with fibromyalgia (a condition characterized by persistent pain that disproportionately affects women). Consistent with Cheng et al [26], there were no gender differences in treatment response. We are unaware of any studies that have explicitly explored gender differences in treatment response in adolescents. Understanding whether gender differences in symptom profiles and treatment response are present during adolescence, when symptoms often first emerge [28], could assist in improving CBT-I outcomes by tailoring them to the needs of the individual’s gender, rather than applying a “one size fits all” approach. This knowledge is particularly important when designing digitally delivered interventions as a face-to-face therapist is not present to adjust the intervention according to the individual’s needs.

The purpose of this study was primarily to investigate possible gender differences in self-reported insomnia symptom severity, sleep quality, and sleep characteristics in adolescents; the secondary purpose was to examine gender differences in CBT-I treatment response in terms of improvements in self-reported insomnia symptoms and sleep quality. Baseline and posttreatment scores on the ISI and Pittsburgh Sleep Quality Index (PSQI) and baseline-only sleep diary variables were compared between girls and boys aged 12-16 years that participated in a published single-group, pre-post pilot trial evaluating the efficacy of a digital CBT-I intervention for adolescent insomnia [29]. Sleep diary variables were included in addition to the PSQI to provide a report of current sleep characteristics (eg, nighttime wakefulness, difficulty falling asleep). Based on the adult literature, we predicted girls would report worse symptom severity, sleep quality, and sleep characteristics consistent with sleep disturbance compared to boys. Based again on the adult literature, we did not anticipate finding significant gender differences in CBT-I treatment response in an adolescent sample.

## Methods

### Participants

This study used data from 49 young people who participated in a pilot trial evaluating Sleep Ninja, a CBT-I smartphone app for young people [29]. Three hundred individuals expressed interest in participating in the Sleep Ninja pilot trial. Of those, 60 reported meeting inclusion criteria and provided written informed consent, along with parental or carer consent, before being formally screened for eligibility. Of these, 10 were not enrolled in the trial, including 4 who did not meet inclusion criteria and 6 who withdrew prior to the trial. Withdrawal reasons included change of mind (n=1), lack of time (n=2), and finding participation a chore (n=1), and 1 person did not provide a reason. Of the remaining 50 participants, 1 was excluded from this study because they did not disclose gender, and 1 boy and 1 girl completed fewer than 6 sleep diaries so were not included in baseline sleep variable comparisons. Gender was assessed via an item on the demographic survey, which asked “What is your gender?”, with possible responses being male, female, or other. Participants were not excluded based on reported gender. Participants were 33 girls and 16 boys aged between 12 and 16 years who met the study inclusion criteria of at least mild insomnia symptoms (operationalized as endorsement of at least one of the first three items on the ISI: difficulty falling asleep, difficulty staying asleep, or waking up too early) and access to a smartphone, internet, and a valid email address.

Thirty-three adolescents completed postintervention assessment. Two were not invited to complete the postintervention assessment because they did not download the Sleep Ninja app. The attrition rates from preintervention to postintervention assessment were similar among boys (6/16, 38% attrition) and girls (10/33, 30% attrition).

### Measures

#### Insomnia Severity Index (ISI)

The ISI is a 7-item self-report measure of insomnia symptoms over the previous 2 weeks that is psychometrically sound [30]. Responses are reported on a Likert scale from 0 to 4, producing total scores of 0-28. Cutoff scores are as follows: 0-7 reflects no clinically significant insomnia, 8-14 indicates subthreshold insomnia, 15-21 suggests moderate severity insomnia, and 22-28 indicates severe insomnia [30]. The ISI was designed for use in adults but has been widely administered to, and validated in, adolescent samples [31-33]. In one adolescent validation study, reliability was strong (Cronbach α=.83), and test-retest reliability was acceptable (r=0.79) [33].

#### Pittsburgh Sleep Quality Index (PSQI)

The PSQI is a 19-item self-report scale that is widely used to assess usual sleep habits and experiences over the preceding month. It has been validated in adolescent samples, with strong internal consistency (α=.72) and test-retest reliability over a 6-week period (r=0.81) [34]. There are 7 subscales, which are sleep quality, sleep latency, sleep duration, habitual sleep efficiency (SE), sleep disturbances, use of sleeping medications, and daytime dysfunction [35]. Each component is scored from 0 (no difficulty) to 3 (severe difficulty), and the components are summed to obtain a Global PSQI score ranging from 0 to 21 [34]. The Global PSQI score was used in this study because it is a valid representation of self-reported sleep quality [21,36,37].

#### Sleep Diary

The 10-item sleep diary was developed by the research team by incorporating the questions from the Consensus Sleep Diary [38], with the addition of 2 questions regarding daytime naps and use of sleep medication. Participants answered 10 questions, which included bedtime, time taken to fall asleep (SOL), number and duration of nighttime awakenings (number of awakenings; NWAK), duration of wakefulness after sleep onset (WASO), time of final awakening, time participants got out of bed for the day, subjective sleep quality, how refreshed participants felt on awakening, duration of any daytime naps, and use of sleep medication. Sleep diary variables obtained via self-report have been found to be consistent with more objective (eg, polysomnography) measures of sleep characteristics [39-41] and were included in this study to provide as objective a report of sleep characteristics as possible in the absence of objective measures. Further details regarding the sleep diary can be found in Werner-Seidler et al [42].

### Intervention: Sleep Ninja

The Sleep Ninja app and the process employed in its design are described in detail by Werner-Seidler et al [42,43]. It is derived from CBT-I and functions as a fully automated smartphone app consisting of six sequential lessons (each taking 5-10 minutes to complete) delivering core CBT-I strategies: psychoeducation, stimulus control, sleep hygiene, and sleep-focused cognitive therapy. The intervention also includes a sleep tracking function, recommended bedtimes based on sleep guidelines, reminders to start a wind-down routine each night, a series of sleep tips, and general information about sleep. The lessons are delivered via a chatbot feature where the sleep ninja acts as a sleep coach. The chatbot feature contains forced choice responses allowing the “chat” to be responsive to the input of the user by personalizing information and recommendations based on the selections and sleep profile recorded by the user. Upon completion of a lesson and 3 nights of sleep tracking (out of a 7-night period), users level up and reach their next “belt.”

### Procedure

This study was conducted with written consent from each participant and their parent or carer, and all procedures were carried out in accordance with the Declaration of Helsinki and approved by the University of New South Wales Human Research Ethics Committee (approval number: HC16702). Participants were recruited through media and social media channels, including the Black Dog Institute’s website and paid Facebook advertisements that targeted potential participants and their parents between April and June 2017. After providing their consent and parental consent via the study website, participants were invited to complete screening questionnaires to verify study eligibility. They then completed demographic and baseline questionnaires (delivered online and described in detail by Werner-Seidler et al [29]): ISI [30] and PSQI [34] to measure symptom severity and sleep quality, respectively, and a 7-day sleep diary to provide a current report of sleep characteristics. We included measures of symptom severity (reflecting symptoms of disorder, including functional impact) and sleep quality (a more general construct related to sleep quality and duration) because, despite typically co-occurring, a subclinical population may experience poor sleep quality in the absence of insomnia symptoms. The Patient Health Questionnaire–Adolescent Version and Generalized Anxiety Disorder–7 were also administered as part of the larger study with outcomes reported by Werner-Seidler et al [29]. Following completion of the sleep diary, participants gained access to Sleep Ninja for 6 weeks before completing the same battery of questionnaires (posttreatment), resulting in a 6-week interval between baseline and posttreatment assessments. Lesson completion was automatically recorded by the app.

### Statistical Analysis

To determine gender differences in symptom severity and sleep quality, two-tailed independent-samples *t* tests were conducted on baseline ISI and Global PSQI scores, respectively. To determine possible differences between girls and boys on sleep diary variables, summary scores for each variable at baseline, including total sleep time (TST), total wake time (TWT), SOL, WASO, NWAK, and SE, were derived by averaging the 7 baseline sleep diary entries and compared using two-tailed independent-samples *t* tests (participants completing fewer than 6 entries were excluded to ensure reliability [44]). Gender differences in demographics between boys and girls were examined using Fisher exact tests.

Since there was a trend in differences in age between boys and girls, though these differences were not significant, post hoc linear regressions were conducted for each sleep outcome, entering gender (coded as 0=male, 1=female) and age as predictors to determine whether gender predicted insomnia symptom severity, sleep quality, and sleep characteristics when controlling for age.

To examine whether gender predicted declines in ISI and PSQI from pretreatment to posttreatment, we conducted hierarchical linear mixed models using SPSS (version 25; IBM Corp) with restricted maximum likelihood estimation. This modelling approach handles missing data by incorporating all available data from each participant into the analysis. Data from the pretreatment and posttreatment time points were grouped by participant. Participant was specified as a random factor, and time, gender, and age were specified as fixed factors. The outcome variables were ISI and PSQI at both time points, with gender as the predictor. Time was coded as 0 for pre and 1 for post, and gender was coded as 0 for male and 1 for female participants. We specified two sets of multilevel models for each outcome variable. In the first model we entered time, gender, and age as predictors, and in the second model we added the two-way interactions between time and gender, time and age, and age and gender. For all models, we specified a random intercept, which allows participants’ mean levels of each outcome to vary. Intraclass correlations (ICCs) were calculated for ISI and PSQI based on intercept-only models. ICC values indicate the amount of variance accounted for by within-person variability. Convention suggests that variables with ICC values greater than 0.10 show sufficient dependency to be analyzed with multilevel modelling [45]. ICCs were 0.42 for ISI and 0.81 for PSQI.

## Results

### Gender Differences in Baseline Symptoms

Baseline score comparisons are presented in Table 1. Boys and girls did not significantly differ in age or city residence; however, more girls than boys were currently using medication and receiving mental health treatment. Girls had significantly higher ISI scores at baseline (*t*_47_=−2.13, *P*=.04; Cohen *d*=0.60), suggesting significantly greater symptom severity compared to boys. Differences in PSQI scores did not reach significance (*t*_47_=−1.90, *P*=.07; *d*=0.57), suggesting negligible differences between girls and boys in sleep quality. Girls reported significantly greater WASO and TWT and significantly reduced TST and sleep efficiency compared to boys (*t*_44_=−2.72, *P*=.01, *d*=0.54; *t*_45_=−2.12, *P*=.04, *d*=0.24; *t*_45_=2.34, *P*=.02, *d*=0.68; *t*_45_=2.28, *P*=.03, *d*=0.30, respectively) but no difference in SOL or NWAK (*P*=.21 and *P*=.16, respectively).

Post hoc linear regressions found that when controlling for age, gender predicted WASO (*t*_44_=2.07, *P*=.04; *f*^2^=0.09), TWT (*t*_45_=2.39, *P*=.02; *f*^2^=0.13), and SE (*t*_45_=−2.18, *P*=.03; *f*^2^=0.11). In contrast, when controlling for age, gender did not predict baseline ISI (*t*_47_=1.42, *P*=.16; *f*^2^=0.04), PSQI (*t*_47_=1.31, *P*=.20’ *f*^2^=0.04), SOL (*t*_45_=−0.57, *P*=.23; *f*^2^=0.04), or NWAK (*t*_45_=0.88, *P*=.39; *f*^2^=0.02) or TST (*t*_45_=1.45, *P*=.15; *f*^2^=0.04). Notably, two variables identified as showing gender differences in the *t* tests, ISI and TST, were not significant predictors in the linear regressions, suggesting baseline gender differences in these variables are accounted for by boys being marginally younger in age.

Of relevance to the current study, there were no differences between boys and girls in the number of Sleep Ninja lessons completed (*P*=.42). Information regarding uptake and adherence to the intervention can be found in Werner-Seidler et al [42].

**Table 1:** Participant characteristics and baseline sleep symptoms.

Characteristic	Male	Female	*P* value
Age (years), mean (SD)	13.70 (1.04)	14.47 (1.51)	.07
Reside in city, n (%)	13 (81)	30 (91)	.38
MH^a^ treatment,^b^ n (%)	0 (0)	13 (39)	.004
Medication use,^c^ n (%)	1 (6)	13 (39)	.02
ISI,^d^ mean (SD)	7.90 (4.63)	9.87 (5.00)	.04
Global PSQI,^e^ mean (SD)	7.40 (3.78)	7.91 (3.90)	.07
SOL,^f,g^ mean (SD)	0.82 (0.75)	0.73 (0.54)	.21
NWAK,^f,h^ mean (SD)	1.50 (2.31)	0.65 (0.69)	.16
WASO,^f,i^ mean (SD)	0.12 (0.18)	0.24 (0.26)	.01
TWT,^f,j^ mean (SD)	1.13 (0.88)	1.40 (1.32)	.04
TST,^f,k^ mean (SD)	8.74 (0.65)	8.14 (1.07)	.02
SE^l^ (%), mean (SD)	88.70 (8.41)	85.80 (10.74)	.03

^a^MH: mental health.

^b^Predominantly psychology.

^c^Predominantly antidepressants, hormonal contraceptives, and melatonin.

^d^ISI: Insomnia Severity Index.

^e^PSQI: Pittsburgh Sleep Quality Index.

^f^In hours, derived by averaging the 7 baseline sleep diary entries.

^g^SOL: sleep onset latency.

^h^NWAK: number of awakenings.

^i^WASO: wake after sleep onset.

^j^TWT: total wake time.

^k^TST: total sleep time.

^l^SE: sleep efficiency.

### Gender Differences in Treatment Response

**Figure 1:**
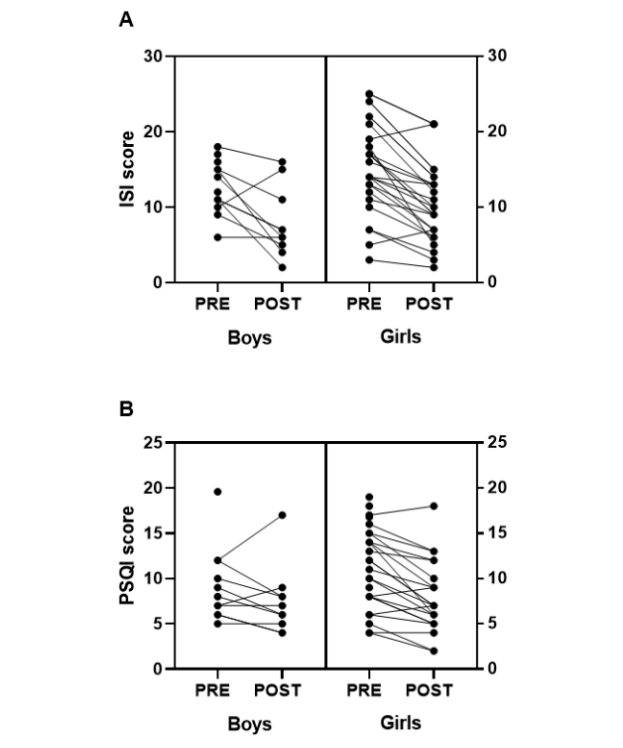
Pretreatment and posttreatment scores for the ISI (A) and PSQI (B) for each participant, grouped by gender. ISI: Insomnia Severity Index. PSQI: Pittsburgh Sleep Quality Index.

Change in ISI and PSQI scores from pretreatment to posttreatment for each participant, grouped by gender, is represented in Figure 1. Results from the mixed-model analysis are presented in Table 2. For insomnia symptom severity, time was a significant predictor of ISI in both models. The coefficient was negative, indicating that scores on the ISI decreased from pretreatment to posttreatment. Gender did not reach significance in either model. The gender by time interaction in model 2 was not significant. Age was not a moderator of any effect.

For sleep quality, time was a significant predictor of PSQI in model 1. The coefficient was negative, indicating that scores on the PSQI decreased from pretreatment to posttreatment. However, time did not remain a significant predictor of PSQI in model 2. Gender did not reach significance in either model. There was a trend in the gender by time interaction in model 2, but it was not significant. Age was not a moderator of any effect.

**Table 2:** Results from the hierarchical linear mixed models.

Characteristic				Parameter estimate		SE		95% CI		*P* value	
**ISI^a^**											
	**Model 1**										
		Intercept	1.94		6.21		−10.55 to 14.43		.76		
		Time	−4.83		0.63		−6.1 to −3.56		<.001		
		Gender	1.6		1.32		−1.06 to 4.26		.23		
		Age	0.8		0.46		−0.13 to 1.73		.09		
	**Model 2**										
		Intercept	0.72		14.61		−28.66 to 30.12		.96		
		Time	−15.44		6.43		−28.5 to −2.38		.02		
		Gender	8.13		16.15		−24.39 to 40.66		.62		
		Age	0.87		1.1		−1.34 to 3.09		.43		
		Time×gender	−1.38		1.4		−4.13 to 1.37		.32		
		Time×age	0.84		0.47		−0.13 to 1.8		.09		
		Gender×age	−0.45		1.2		−2.87 to 1.97		.71		
**PSQI^b^**											
	**Model 1**										
		Intercept	1.25		5.82		−10.49 to 12.99		.83		
		Time	−2.13		0.45		−3.01 to −1.21		<.001		
		Gender	1.02		1.24		−1.43 to 3.57		.39		
		Age	0.61		0.43		−2.66 to 1.48		.17		
	**Model 2**										
		Intercept	8.87		13.63		−18.61 to 36.34		.52		
		Time	−2.06		4.58		−11.42 to 7.29		.66		
		Gender	−7.77		15.15		−38.32 to 22.78		.61		
		Age	0.004		1.03		−2.06 to 2.07		>.99		
		Time×gender	−1.82		0.96		−3.79 to 0.16		.07		
		Time×age	0.08		0.34		−0.6 to 0.77		.80		
		Gender×age	0.71		1.13		−1.57 to 2.98		.54		

^a^ISI: Insomnia Severity Index.

^b^PSQI: Pittsburgh Sleep Quality Index.

## Discussion

This study compared self-reported insomnia symptom severity, sleep quality, sleep characteristics, and CBT-I treatment response between adolescent girls and boys with at least mild symptoms of insomnia. The results supported some of our predictions by showing that girls more often than boys reported sleep characteristics consistent with disturbed sleep, including increased nighttime wakefulness and reduced sleep efficiency. However, contrary to predictions, we found no differences in self-reported sleep quality on the PSQI or in difficulty falling asleep, and gender differences in symptom severity and total sleep duration were accounted for by boys being marginally younger in age. It is not immediately clear why girls reported sleep characteristics consistent with disturbed sleep but not poorer sleep quality on the PSQI or insomnia symptoms on the ISI (when controlling for age) compared to boys. One possible explanation is the nonclinical sample examined in the current study. Individuals with milder symptoms may experience greater symptom fluctuation consistent with individuals in the early stage of disorder development (prodromal phase; eg, [46]), which may be more effectively captured in state-based measures (eg, daily sleep diary) compared to trait-based measures reliant on self-report (eg, PSQI and ISI measure sleep parameters over the past 4 and 2 weeks, respectively).

Increased wakefulness after sleep onset in girls in the current study is consistent with previous reports showing women and adolescent girls more often report sleep characteristics consistent with increased nighttime wakefulness [4,18]. However, unlike these previous studies, we did not specifically find that girls reported more difficulty falling asleep. The previous studies were conducted in adults and in a cohort of adolescents with an average age of 17 years (compared to 14.5 years in the current study), which might account for these differences. Regardless, our results indicate that gender differences in sleep characteristics in older adolescents and adults are at least partially present in younger adolescents (in addition to reduced sleep efficiency in girls). One possible explanation for increased sleep disturbance in girls is the propensity for adolescent and adult females to engage in unhelpful cognitive processes, such as rumination and worry, relative to males [47,48]. Rumination and worry play a central role in sleep disturbance in a range of contexts, including in individuals with sleep disturbances [49], individuals with other mental disorders [50], and healthy individuals [51]. For example, Harvey’s [52] cognitive model of insomnia postulates that excessive worry about sleep and the consequences of poor-quality sleep triggers autonomic arousal, emotional distress, and excessive monitoring of threats to sleep quality (eg, indicators of insufficient sleep or poor functioning during the day), which ultimately results in real deficits in sleep. Thus, established gender differences in worry make these factors that are critical to the development and maintenance of sleep disturbance good candidates to explain gender differences shown in this study. Future studies could employ measures of rumination and worry alongside sleep measures to determine the extent to which they moderate gender differences in symptoms.

Adolescence has been identified as a period of substantial change in terms of biopsychosocial development, which has been attributed to changing sleep patterns in both genders in this age group [13]. However, pubertal maturation seems to be particularly significant for girls in regard to sleep. Several studies have found that in girls, but not boys, the onset of pubertal maturation is a significant risk factor in the development of insomnia and is associated with increased symptom severity [16,17]. Therefore, another possible mechanism underlying gender differences in pretreatment symptoms is hormonal fluctuations associated with the onset of puberty in girls. The menstrual cycle is a major source of hormonal fluctuations in estradiol and progesterone over a 4-week period. Estradiol and progesterone levels are initially low (follicular phase), with a midcycle peak in estradiol indicating ovulation, followed by a rise in both estradiol and progesterone during the second half of the cycle before declining to the initial low levels (luteal phase). In support of this notion, adult women self-report poorer sleep quality during periods of hormonal flux, including the luteal phase of the menstrual cycle, perinatal period and menopause [17,18,53]; however, the association between sex hormones and more objectively measured sleep variables (eg, via polysomnography) is more contentious [54]. The nature of the association between pubertal maturation and insomnia prevalence and disturbed sleep characteristics, including the potential role of fluctuating sex hormones, requires further examination.

Despite pretreatment differences in symptom severity, boys and girls responded comparably to CBT-I, as evidenced by comparable reductions in symptom severity. On the one hand, it is encouraging that despite greater pretreatment symptom severity, girls gain the same benefits from treatment as boys. On the other hand, equivalent treatment-elicited symptom reduction would suggest gender differences in symptom severity at baseline are maintained posttreatment, which we speculate may render girls more vulnerable to relapse than boys. More work in this area is required to determine if differences remain between boys’ and girls’ posttreatment symptom levels. We were unable to adequately analyze this here due to participant attrition and therefore an incomparable sample at baseline and posttreatment. Future studies could also examine if there are gender differences in responsiveness to certain treatment elements. For example, as girls engage in more rumination and worry [47,48], which are perpetuating features of insomnia (as discussed above), they may benefit from extending the cognitive aspect of CBT-I to reduce the frequency of these cognitive maintaining factors and thus limit ongoing sleep disturbance. These ideas require further investigation to refine gender-specific delivery of CBT-I.

Our study had several limitations, namely the lack of a nontreatment control group to exclude the possibility that participants responded to some nontherapeutic aspect of CBT-I. Our study also relied on subjectively reported sleep diary variables. The inclusion of polysomnography or actigraphy would help establish gender differences in objectively measured sleep parameters. However, the presence of gender differences on some sleep characteristics but not others in the current study suggests that our results are not merely driven by a bias for girls to overreport (or boys to underreport) symptoms in general. A disproportionate number of girls participated in this study. This is consistent with male to female participant ratios reported in similar studies evaluating CBT-I in adolescents that use a range of recruitment strategies (eg, community recruitment via a study website [23] and outpatient mental health clinics [55]). This suggests gender disparity in participation is representative of the increased prevalence of sleep problems in girls compared to boys in the community. The small sample size, particularly the small sample of boys, should also be taken into consideration when interpreting the current results. It is possible that a lack of power to detect significant effects concealed true differences between boys and girls in treatment response (particularly in reductions in sleep quality, in which there was a trend, though it was not statistically significant) and baseline sleep characteristics. Future studies could ensure adequate power to detect possible gender differences by recruiting larger samples with equal numbers of boys and girls.

A further limitation is that the current study did not assess pubertal maturation, which has previously been shown to be associated with gender differences in insomnia [16]. This information would have provided greater insight into the potential role of hormonal fluctuation in girls’ experience of sleep disturbance. Despite these limitations, our study has demonstrated the importance of continued investigation into understanding the nature of gender differences in insomnia and sleep disturbance in adolescents. This knowledge could be applied in future research aimed at refining insomnia interventions according to the unique needs of each gender, especially in digital interventions as this could inform onboarding processes to facilitate the personalization of the intervention to the benefit of both genders.

## Abbreviations

CBT-I: cognitive behavioral therapy for insomnia
ICC: intraclass correlation
ISI: Insomnia Severity Index
NWAK: number of awakenings
PSQI: Pittsburgh Sleep Quality Index
SE: sleep efficiency
SOL: sleep onset latency
TST: total sleep time
TWT: total wake time
WASO: wake after sleep onset
